# Whole genome sequencing analysis of *Candida glabrata* isolates collected from patients with selected drug-resistant candidiasis hospitalized in Eastern Poland

**DOI:** 10.1007/s12223-025-01298-w

**Published:** 2025-07-14

**Authors:** Sebastian Kubica, Weronika Szulińska, Celina Kruszniewska-Rajs, Magdalena Kimsa-Dudek, Alina Olender, Agnieszka Bogut, Magdalena Szukała, Wojciech Dąbrowski, Daniel Pietrzak, Mariusz Gagoś, Joanna Magdalena Gola

**Affiliations:** 1 Department of Molecular Biology, Faculty of Pharmaceutical Sciences in Sosnowiec, Medical University of Silesia, Katowice, 40-055 Poland; 2 Chair and Department of Medical Microbiology, Medical University of Lublin, 20-093 Lublin, Poland; 3 First Department of Anaesthesiology and Intensive Therapy, Medical University of Lublin, 20-090 Lublin, Poland; 4 Second Department of Anaesthesiology and Intensive Therapy, Medical University of Lublin, 20-400 Lublin, Poland; 5 Department of Cell Biology, Maria Curie-Sklodowska University, 20-033 Lublin, Poland

**Keywords:** *Candida glabrata*, Candidiasis, Eastern Poland, Whole genome sequencing

## Abstract

The epidemiology data for candidiasis indicate an increase in *Candida glabrata* infections. Moreover, several reports have shown an increasing number of drug-resistant cases of these infections. The source of drug resistance can often be traced to genetic mutations in genes related to a drug’s mechanism of action. Therefore, we conducted whole genome sequencing of several drug-resistant isolates of *Candida glabrata* collected from patients hospitalized in Eastern Poland to assess whether mutations in selected genes correlated with susceptibility analysis results. The fungal species from patient samples were identified, and the isolated *Candida glabrata* were subjected to antifungal drug susceptibility testing. The results were interpreted according to the EUCAST and CLSI recommendations. Susceptibility to 5-flucytosine was assessed using the ATB FUNGUS kit. Libraries were prepared according to the NEXTERA XT DNA Library Prep and subsequently sequenced. The outcomes indicated common resistance to two of the three analyzed echinocandins, as well as two cases of simultaneous resistance to echinocandins and selected azole-based drugs. We detected several previously reported mutations in selected resistance-related genes, as well as five that are first described here: *ERG5* (M267I), *ERG6* (R57K), *PDH1* (K438Q, V434I, F600V, V1192S), *FCY1* (M129T), and *FCY2* (I384F). Neither of the identified nonsynonymous mutations was correlated with the drug resistance demonstrated in the susceptibility testing. Furthermore, we can exclude the possibility of acquired drug resistance, thereby raising questions about the possibility of unknown mechanisms of resistance to azole-based and echinocandin drugs.

## Introduction

Candidiasis in the third decade of the twenty-first century remains a significant condition affecting individuals worldwide. The epidemiology of infections caused by *Candida* spp. has changed over the years (Arendrup et al. 2023). In the 1980s–1990s, *Candida albicans* was considered the most common fungus; for example, it was responsible for up to 73% of all fungal infections observed in Rigshospitalet in Copenhagen (Nielsen et al. 1991) and for 80% in the University of Iowa Hospitals and Clinics (Wenzel 1995). These infections were predominantly treated with amphotericin B and 5-flucytosine, as very few *Candida albicans* strains were resistant.

On the contrary, nationwide surveillance programs conducted in France and Denmark during the 2010s–2020s reported that *Candida albicans* accounted for 55.6% (Bretagne et al. 2022) and 42.1% (Risum et al. 2021) of all noted infections, respectively. Simultaneously, the same surveillance programs revealed an increase in infections caused by *Candida glabrata* (*C. glabrata*). In France, *C. glabrata* was isolated from 18.5% (Bretagne et al. 2022) of patients with yeast fungemia, while in Denmark, this percentage was 32.31% (Risum et al. 2021). Furthermore, numerous isolated strains have been reported to demonstrate resistance to azole drugs and to have acquired echinocandin resistance. These findings have necessitated the development of novel antifungal drugs and a thorough examination of the mechanisms of acquired resistance.

In our research, we have decided to focus on *C. glabrata* due to its increasing occurrence in fungal infections and because its resistance to commonly used antifungal drugs poses a threat to patient health. *C. glabrata* (also known as *Torulopsis glabrata* or *Nakaseomyces glabratus*) was historically characterized as a safe saprotrophic yeast present in healthy microflora, predominantly in the oral cavity, and was considered capable of causing pathologic infections only under certain conditions (Haley 1961; Stenderup and Pedersen 1962). However, *C. glabrata* is currently regarded as the second most prevalent pathogen in fungal infections in the USA, the UK, Northern Europe, and Australia, although *Candida parapsilosis* is more common in Southern Europe (Colombo et al. 2017). *C. glabrata* is innately more resistant to azole-based drugs that target fungal cell membranes because it produces various adhesins that facilitate adherence to both biotic and abiotic surfaces and promote the formation of biofilm, which acts as an additional protective barrier (Timmermans et al. 2018). However, genetic mechanisms also underlie fungal resistance to common antifungal drugs.

**Fig. 1:**
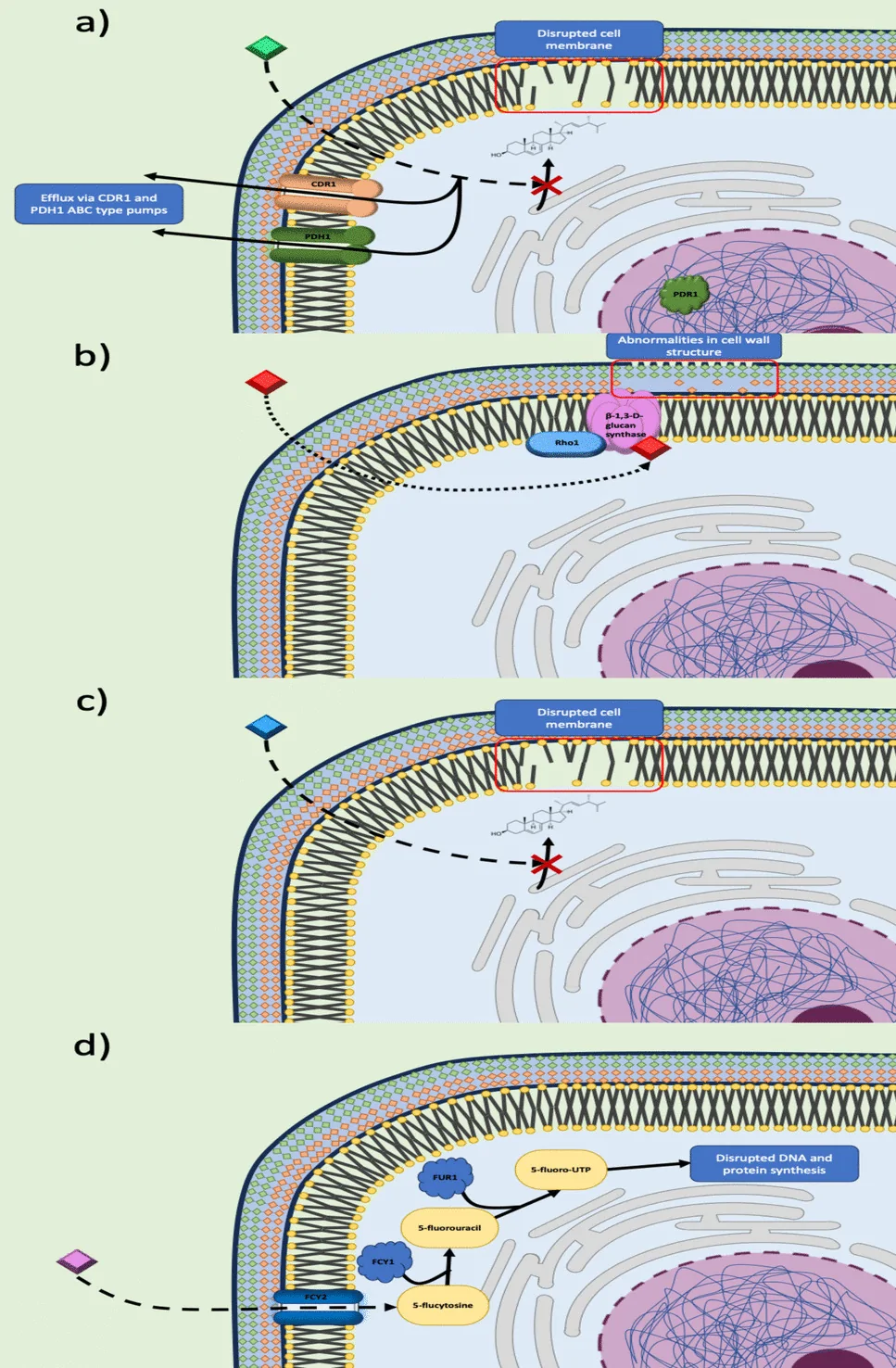
Mechanisms of action of selected antifungal drugs: **a** Azole-based drugs enter the cell and disrupt the synthesis of ergosterol, which leads to a disruption of the cell membrane. Resistance is mediated through efflux via the ABC-type transporters CDR1 and PDH1. PDR1 is often related to the overexpression of these transporters (Navarro-Rodríguez et al. 2020). **b** Echinocandins, after entering fungal cells, act as uncompetitive inhibitors of β−1,3-D-glucan synthase. The disruption of this enzyme activity results in abnormalities in the structure of the cell wall. Resistance to echinocandins is associated with mutations in the FKS1 and FKS2 subunits of β−1,3-D-glucan synthase (Szymański et al. 2022). **c** Amphotericin B acts similarly to azole-based drugs by inhibiting the synthesis of ergosterol and disrupting the cell membrane. Resistance mechanisms are associated with mutations in the *ERG2*, *ERG3*, *ERG5*, *ERG6*, and *ERG11* genes, which encode enzymes relevant to ergosterol biosynthesis (Gray et al. 2012). **d** 5-Flucytosine enters the cell via a cytosine permease (FCY2) and is then converted to 5-fluorouracil by cytosine deaminase (FCY1). The 5-fluorouracil is subsequently converted by uracil phosphoribosyltransferase (FUR1) to 5-fluoro-UTP, which takes part in the disruption of DNA and protein synthesis. Resistance to 5-flucytosine is often caused by mutations in the *FCY1*, *FCY2*, and *FUR1* genes (Gopinathan et al. 2013)

As presented in Fig. 1, acquired azole resistance is often mediated by the overexpression of the *CDR1* and *CDR2* (*PDH1*) genes that encode the ABC transporters involved in drug efflux. This overexpression is commonly associated with mutations in the *PDR1* gene, which encodes a transcription factor essential for the expression of the *CDR1* and *CDR2* genes (Ferrari et al. 2009; Tsai et al. 2010). Conversely, low susceptibility to echinocandins is related to *FKS* gene mutations, which are more prevalent among patients previously treated with echinocandins (Szymański et al. 2022; Beyda et al. 2014). However, echinocandin resistance may also occur even in patients with a wild-type *FKS* genotype (Aldejohann et al. 2021), meaning that another mechanism must be responsible for lowered susceptibility. At present, among the commonly used antifungal drugs, only amphotericin B and 5-flucytosine remain viable treatments against the emerging threat of *C. glabrata-*initiated candidiasis.

In the present study, based on the available and relevant literature, we selected genes related to drug resistance, as summarized in Table 1, and assessed mutations occurring in samples of *C. glabrata* harvested from patients infected with drug-resistant strains. The imminent threat of a rising proportion of candidiasis caused by *C. glabrata* highlights the importance of this research. Although several similar studies have recently reported whole genome sequencing (WGS) of *C.* strains isolated from patients diagnosed with fungemia (Biswas et al. 2017, 2018; Lim et al. 2023; Misas et al. 2024), our research differs in its examination of resistant strains gathered from patients before any antifungal treatment. Our results obtained from WGS are inconclusive regarding any association between mutations in selected drug resistance-related genes and a reduction in the effectiveness of antifungal drugs. Antifungal drug resistance among clinical isolates in Poland has been addressed in several studies (Paluchowska et al. 2014; Szweda et al. 2015; Szymankiewicz et al. 2021); however, to our knowledge, this is the first research involving WGS in search of genetic mutations underlying this resistance.

**Table 1:** Summary of selected drug resistance-related *C. glabrata* genes

Drug resistance	Gene	Chromosome accession number	Chromosome localization	Source
Azoles	*CDR1*	CR380959.2	213,414–217,913	(Tsai et al. 2010; Yoo et al. 2010; Navarro-Rodríguez et al. 2020)
	*PDH1*	CR380952.1	284,437–289,065	
	*ERG3*	CR380952.1	200,772–201,866	
	*ERG11*	CR380951.2	432,898–434,499	
	*PDR1*	CR380947.2	62,205–65,528	
Echinocandins	*FKS1*	CR380953.1	107,061–112,652	(Park et al. 2005; Perlin 2007; Cleary et al. 2008)
	*FKS2*	CR380957.2	375,102–380,795	
Amphotericin B	*ERG2*	CR380958.2	1,157,339–1,158,013	(Vandeputte et al. 2008; Martel et al. 2010b, a; Hull et al. 2012)
	*ERG3*	CR380952.1	200,772–201,866	
	*ERG5*	CR380959.2	775,570–777,177	
	*ERG6*	CR380954.1	449,754–450,872	
	*ERG11*	CR380951.2	432,898–434,499	
5-Flucytosine	*FCY1*	CR380950.1	176,628–177,095	(Edlind and Katiyar 2010; Vandeputte et al. 2011)
	*FCY2*	CR380956.2	304,828–306,375	
	*FUR1*	CR380954.1	890,276–890,926	

## Materials and methods

### Sample characteristics

Prior to antifungal treatment, samples of various origins were collected from patients diagnosed with fungal infections and hospitalized in the Department of Anaesthesiology and Intensive Therapy in Lublin. Candidiasis was confirmed in 83 cases of fungemia, and all isolates derived from these 83 patients were further analyzed using WGS. To further narrow the scope of this study, we focused on the 10 *C. glabrata* isolates found in the analysis. A detailed description of each phase of the research is presented in the subsequent “Materials and methods” sections.

### Sample isolation

The *C. glabrata* isolates used in this study were cultured from 10 patients. Clinical specimens included blood, sputum, pus, urine, and throat swabs. The samples were cultured on Sabouraud (bioMerieux) medium for up to 5 days at 30 °C.

### Identification of fungal species

Microscopy preparations were stained using the Gram method. Upon light microscopy confirmation of a yeast presence, the fungus was phenotypically identified using commercial biochemical tests (ID32 [bioMérieux] and the VITEK 2-YST yeast identification card using the VITEC 2com [bioMérieux] device) according to the manufacturer’s recommendations.

### Antifungal susceptibility testing

The susceptibility of *C. glabrata* isolates to the following antifungal drugs was determined: amphotericin B, the azoles fluconazole, itraconazole, voriconazole, isavuconazole, and posaconazole, and the echinocandins caspofungin, micafungin, and anidulafungin. The determination of the minimum inhibitory concentration (MIC) for the tested drugs was performed using MIC test strips (Liofilichem) on Roswell Park Memorial Institute (RPMI) agar (bioMerieux) and 0.5 McFarland inoculum suspension in physiological saline, incubated at 35 °C. The MIC was read after 24 h (confirmed after 48 h). The MIC results were determined according to the manufacturer’s recommendations (Liofilichem: amphotericin B, value at complete growth inhibition; azoles, at the first point of significant inhibition/significant decrease in growth density (80% inhibition rule); echinocandins, at the point of significant inhibition (i.e., 80% inhibition). The results were interpreted according to the EUCAST (The European Committee on Antimicrobial Susceptibility Testing) (2023) and CLSI (Clinical and Laboratory Standards Institute) (2022) recommendations. The antibiotic susceptibility tests were performed in duplicate, and the final results were reported as the average of two MIC determinations. The reference strain *Candida parapsilosis* ATCC 22019 was used for quality control of the MIC value determination in accordance with the EUCAST (2022) recommendations. The ATB FUNGUS kit (bioMerieux) was also used to determine sensitivity to 5-flucitosine, and the test was performed according to the manufacturer’s instructions.

### Isolation of *C. glabrata* genomic DNA

Genomic DNA was isolated from broth cultures of the tested *C. glabrata* isolates using the Genomic Mini AX Yeast kit (A&A Biotechnology, Gdynia, Poland) according to the manufacturer’s instructions. The concentration and quality of the isolated DNA samples were determined using a BioTek Synergy LX reader (Agilent, Santa Clara, CA, USA).

### WGS and bioinformatics analysis

Isolated DNA was quantified, and its quality was assessed using a Quantus fluorimeter and the QuantiFluor ONE dsDNA System (Promega, Madison, WA, USA). Dual-indexed, paired-end libraries were prepared from 1 ng of genomic DNA according to the manufacturer’s instructions using the NEXTERA XT DNA Library Prep (Illumina, San Diego, CA, USA). Briefly, the procedure included tagmentation of genomic DNA, amplification of the tagmented DNA, and library clean-up, normalization, and pooling. The quality of the libraries was assessed using an Agilent Technology 2100 Bioanalyzer (Agilent, Santa Clara, CA, USA) and the High Sensitivity DNA kit (Agilent, Santa Clara, CA, USA).

The libraries were subsequently sequenced using the Illumina NextSeq 550 platform (Illumina, San Diego, CA, USA) in accordance with the manufacturer’s instructions. The generated data were then subjected to bioinformatics analysis. First, the raw data quality (fastq.gz) was assessed using the FastQC High Throughput Sequence QC (version 0.12.1) (Babraham Bioinformatics 2023). The data were then trimmed using Trimmomatic (package version: 0.39) (Bolger et al. 2014). The trimmed sequences were then aligned to the *C. glabrata* CBS138 reference genome, accessed from the NCBI database (Genome assembly ASM1011175v1) using the Burrows-Wheeler Aligner (BWA) (version 0.7.18-r1243-dirty) (Li and Durbin 2009). Duplicate marking, filtration, and conversion to the BAM file were performed using Samtools (version 1.20) (Li et al. 2009). Using freebayes (version 1.3.6) (Garrison and Marth 2012), variants were called and subsequently filtered with bcftools (version 1.20) (2024). Low-quality variants were removed using bcftools (version 1.20).

The obtained results were filtered by compiling a list of genes associated with antifungal resistance based on data published in recent articles. We identified 13 genes that were crucial for resistance to the tested antifungal drugs (Table 1). Based on the chromosomal localization of these genes, we translated the DNA sequence into a potential polypeptide chain using the Expasy translation tool (Gasteiger et al. 2003). These amino acid sequences were then compared, using the blast algorithm (Altschul et al. 1997), to reference sequences accessed from the NCBI Protein database to reveal nonsynonymous mutations.

## Results

WGS analysis yielded an average of 5,027,903.1 reads per sample, with a mapping rate of 98.4% to the reference genome (CBS138) and an average coverage of 101.77X. Low-quality results were rejected during trimming (average trimming rate = 11.4%), leading to 91% of all reads having a quality score greater than 30. Variant calling resulted in an average of 61,738 mutations (5016 insertion/deletion, INDEL; 56,722 single nucleotide polymorphisms, SNP) detected per sample, of which 16.1% were nonsynonymous. Apart from INDEL and single nucleotide polymorphism (SNP) mutation types, an average of 5059 multiple nucleotide polymorphisms (MNPs) were noted. Nonsynonymous mutations were further filtered according to previously selected genes related to drug resistance. This filtering resulted in an average of six mutations in these genes. Table 2 summarizes the mutations detected in 10 samples using WGS.

**Table 2:** Summary of mutations detected in 10 samples of *C. glabrata* using WGS

Isolate No	Total mutations					Nonsynonymous mutations			Nonsynonymous mutations in the resistant genes		
	INDEL	SNP	MNP	Other	Total	INDEL	SNP	Total	INDEL	SNP	Total
3	4466	50,351	4309	1260	60,195	777	7521	8298	0	6	6
5	4566	49,530	4386	1271	59,533	836	7247	8083	0	3	3
6	5829	65,098	6113	1699	78,412	1093	9135	10,228	0	7	7
14	4313	49,838	4164	1217	59,364	762	7452	8214	0	6	6
25	5865	67,142	6281	1673	80,633	1139	9547	10,686	1	4	5
28	4726	51,321	4482	1345	61,662	790	7535	8325	0	6	6
29	4712	51,175	4469	1330	61,461	780	7520	8300	0	8	8
47	4330	49,997	4230	1215	59,599	756	7494	8250	0	6	6
60	5840	67,138	6262	1680	80,591	1150	9544	10,694	1	4	5
80	5508	65,632	5890	1692	78,445	1089	9169	10,258	0	11	11
Total	50,155	567,222	50,586	14,382	679,895	9172	82,164	91,336	2	61	63
Average	5016	56,722	5059	1438	67,990	917	8216	9134	0	6	6
SD	628.42	7819.75	890.32	206.13	9467.45	165.87	936.11	1099.27	0.40	2.17	2.00

A comparison of the nonsynonymous mutations in genes related to antifungal drug resistance detected in the *C. glabrata* isolates with the MIC results (Table 3) revealed that all samples bore identical mutations in the following genes: *ERG2* (I207V), *PDH1* (E839D), and *PDR1* (V91I, L98S). Moreover, in *PDR1*, an interesting relationship was noted, as eight samples had T143P and S76P substitutions, and the remaining two had D243N substitutions, but these mutations never occurred together in a single sample. Frequently occurring mutations were also detected in *ERG2* (L60F), *CDR1* (H58Y), and *FKS2* (E78D, T928P). Some of the mutations of lower frequency (≥ 30%) detected in resistance-related genes in our *C. glabrata* isolates have not been reported previously. These include *ERG5* (M267I), *ERG6* (R57K), *PDH1* (K438Q, V434I, F600V, V1192S), *FCY1* (M129T), and *FCY2* (I384F).

**Table 3:** MIC values and nonsynonymous mutations in the analyzed *C. glabrata* isolates

Isolate no	3	5	6	14	25	28	29	47	60		80
Source	Blood	Sputum	Throat swab	Sputum	Pus	Urine	Blood	Sputum	Sputum		Sputum
**MIC (mg/L)**											
Amphotericin B	0.38	0.19	0.25	0.75	0.5	0.38	0.25	0.25	0.25		0.38
Fluconazole	2.0	4.0	8.0	24.0^a^	24.0^a^	6.0	4.0	0.5	8.0		1.0
Itraconazole	** > 32**	1.0	2.0	** > 32**	** > 32**	6.0	** > 32**	1.5	4.0		0.125
Voriconazole	0.125	0.19	0.125	1.5	3.0	8.0	0.5	0.38	0.25		0.25
Isavuconazole	0.094	0.25	0.125	1.5	1.0	0.25	0.5	0.19	0.25		0.38
Posaconazole	4.0	2.0	1.5	** > 32**	** > 32**	3.0	12.0	1.5	4.0		3.0
Caspofungin	0.75^b^	0.38	0.25	1.0^b^	0.75^b^	0.75^b^	0.5^b^	0.5^b^	0.5^b^		0.5^b^
Micafungin	0.006	0.004	0.006	0.008	0.006	0.006	0.006	0.006	0.016		0.012
Anidulafungin	0.094^a^	0.006	0.047	0.094^a^	0.125^a^	0.064^a^	0.012	0.064^a^	0.125^a^		0.047
**Nonsynonymous mutations**											
*ERG2*	L60F I207V	I207V	I207V	L60F I207V	I207V	L60F I207V	L60F I207V	L60F I207V	I207V	I207V	
*ERG5*							M267I^c^				
*ERG6*										R57K^c^	
*CDR1*	H58Y			H58Y		H58Y	H58Y	H58Y		H58Y	
*PDH1*	E839D	E839D	K438Q^c^ V434I^c^ E839D	E839D	K438Q^c^ E839D	E839D	V812A^c^ E839D	E839D	K438Q^c^ E839D	F600V^c^ E839D A1192S^c^ T1530K	
*PDR1*	S76P V91I L98S T143P	S76P V91I L98S T143P	S76P V91 L98S T143P	S76P V91I L98S T143P	V91I L98S D243N	S76P V91I L98S T143P	S76P V91I L98S T143P	S76P V91I L98S T143P	V91I L98S D243N	S76P V91I L98S T143P	
*FKS1*					G14S				G14S		
*FKS2*	E78D T928P^c^			E78D	T928P^c^	E78D T928P^c^	E78D T928P^c^	E78D T928P^c^	T928P^c^		
*FCY1*			M129T^c^								
*FCY2*			I384F^c^								

^a^EUCAST (fluconazole R>16; anidulafungin R≥0.06); ^b^CLSI (caspofungin R≥0.5); values in **boldface** (MIC>32) are indicative of resistance with the lack of recommendations for the result interpretation; ^c^mutations that have not been annotated to this date

The results for susceptibility to antifungal drugs indicated a rather common resistance to two of the three analyzed echinocandin drugs. We determined that 80% of the *C. glabrata* strains were resistant to caspofungin, while 70% exhibited resistance to anidulafungin. Among the three tested echinocandin drugs, only micafungin proved effective at inhibiting fungal growth. In contrast, resistance to selected substances from the azole group was significantly less common. Only two strains were unaffected by fluconazole, itraconazole, and posaconazole. One more strain was resistant to itraconazole, but the remaining azole drugs effectively limited its growth. None of the isolated strains exhibited resistance to voriconazole or isavuconazole. Moreover, amphotericin B and 5-flucytosine (ATB FUNGUS test results not shown) successfully limited the growth of all analyzed strains.

## Discussion

Amphotericin B is a potent antifungal drug commonly employed as an alternative to azoles and echinocandins. Its antifungal effects are determined by two separate mechanisms (Gray et al. 2012): one involves the binding of ergosterol, a lipid that is crucial in the process of yeast cell membrane formation. The impairment of this process leads to severe physiological disorders that may lead to the death of yeast cells. The second mechanism involves channel-mediated membrane permeabilization, which ultimately results in the lysis of the yeast cell. *ERG2*, *ERG3*, *ERG5*, *ERG6*, and *ERG11* encode proteins involved in ergosterol biosynthesis, and mutations in these genes have been previously reported to alter yeast susceptibility to amphotericin B (Hull et al. 2012; Ahmad et al. 2019). Our WGS results confirmed previous conclusions about the I207V alteration in the ERG2 protein, which is considered to be a genetic polymorphism present both in wild-type and resistant strains of *C. glabrata* (Hull et al. 2012; Ahmad et al. 2019). Similarly, in our research, the presence of this nonsynonymous mutation in all sequenced samples did not affect susceptibility to amphotericin B. Another relatively common mutation identified in *ERG2*, resulting in L60F alteration in our samples, has been previously described as having no effect on amphotericin B resistance (Hull et al. 2012). Among the remaining selected genes involved in amphotericin B resistance mechanisms, we identified novel mutations resulting in M267I (sample 29) and R57K (sample 80) alterations in *ERG5* and *ERG6* expression products. However, these mutations did not alter susceptibility to amphotericin B, as all our isolates proved vulnerable. No mutations were detected in *ERG3* or *ERG11*.

The *CDR1*, *PDH1*, and *SNQ2* genes encode ABC transporters involved in drug efflux, and their overexpression often leads to acquired azole resistance. Their significance was assessed by Whaley et al. (2018), who found that overexpression of *CDR1* was the primary factor for azole resistance, even though all three transporters are involved in this phenomenon. In the present study, alterations in genetic expression levels were not evaluated, but we have identified several mutations located in the genes encoding CDR1, PDH1, and PDR1 transcription factors. The H58Y alteration in the CDR1 protein, while relatively common, did not affect susceptibility to selected azole-based drugs, as it was present in both our resistant and vulnerable *C. glabrata* strains. Lim et al. also classified H58Y as having no involvement in azole resistance (Lim et al. 2023) and pointed out that alterations in E839D and T1530K commonly occurred and did not affect azole resistance, as confirmed by our results.

Other nonsynonymous mutations in *PDH1* that resulted in K438Q, V434I, V812A, F600V, and A1192S have not been previously described. All of these amino acid alterations are located outside ABC transporter domains 1 or 2, based on the UniProt data. None of them seems to be particularly relevant in the context of azole-based drug resistance. All of our isolates had the same nonsynonymous PDR1 mutations V91I and L98S. Eight of them also had S76P and T143P substitutions, and the remaining two had D243N substitutions. We suspect that our results indicate the presence of genetic polymorphism in the *PDR1* sequence, resulting in strains with S76P and T143P or strains with D243N, but all of these alterations are present in both resistant and susceptible strains. Therefore, we suspect that they do not influence azole-based drug resistance mechanisms, confirming the conclusions drawn by Lim et al. (2023) and Ceballos-Garzon et al. (2022).

The fungal cell wall is composed of numerous components, one of which is β−1,3-D-glucan. Echinocandins act as inhibitors of β−1,3-D-glucan synthase; therefore, they contribute to the instability of the fungal cell wall, leading to the death of the fungal cell (Szymański et al. 2022). This mechanism of action is particularly effective because glucan is not a component of plant or animal cells; thus, the use of echinocandins in antifungal treatment remains safe. Echinocandin resistance is linked to mutations in the *FKS1* and *FKS2* genes, which encode subunits of β−1,3-D-glucan synthase (Park et al. 2005; Perlin 2007; Cleary et al. 2008). G14S substitution in FKS1 was previously reported by Ceballos-Garzon et al. (2022) as not associated with echinocandin resistance. Our results indicate that G14S substitution was present in two strains that showed resistance to caspofungin and anidulafungin treatment, whereas other resistant strains lacked it. Therefore, we can confirm that it is not involved in echinocandin resistance. Amino acid alterations in FKS2 identified in our strains were also located outside of mutation hotspots. E78D and T926P were previously described in samples from global surveillance studies reported by Castanheira et al. (2014) or Ceballos-Garzon et al. (2022) and were classified as uninvolved in resistance mechanisms. We have detected E78D and T928P (highly similar to T926P) substitutions in several *C. glabrata* isolates and can confirm that their presence does not correlate with resistance to selected echinocandins.

Resistance to 5-flucytosine treatment is primarily attributed to mutations in *FCY2*, *FCY1*, and *FUR1*, which encode cytosine permease, cytosine deaminase, and uracil phosphoribosyltransferase, respectively (Edlind and Katiyar 2010). 5-Flucytosine enters fungal cells via cytosine permease (Paluszynski et al. 2006) and is subsequently converted to 5-fluorouracil by cytosine deaminase (Polak and Scholer 1975) and then modified to 5-fluoro-UMP by phosphoribosyltransferase. The presence of 5-fluoro-UMP results in the disruption of DNA and enables conversion to 5-fluoro-UTP, which is incorporated into RNA in place of UTP, leading to the disruption of protein biosynthesis (Gopinathan et al. 2013). Mammalian cells lack cytosine deaminase; therefore, 5-flucytosine is considered safe, even though bacteria residing in the gut can convert 5-flucytosine to toxic 5-fluorouracil (Vermes et al. 2000). None of the mutations in genes encoding the proteins relevant to the 5-flucytosine mechanism of antifungal activity was detected in any of our isolates. In isolate 3, we detected substitutions M129T in FCY1 and I384F in FCY2 that have not been reported before, but they did not provide immunity to 5-flucytosine, as this strain was as susceptible as the others (data not shown).

In conclusion, in our study, we have isolated ten strains of *C. glabrata* from patients hospitalized due to fungemia. Two strains can be classified as resistant to both azole-based drugs and selected echinocandins. The causes of this multidrug resistance could not be traced to mutations in genes considered relevant to azole or echinocandin resistance. Moreover, as the patients had not been treated prior to the collection of specimens, we can exclude the possibility of acquired resistance. Our results raise the question of whether other mechanisms are responsible for this resistance.

Another notion worth pointing out is that *C. glabrata* was isolated from various samples obtained from patients with fungemia, meaning that *C. glabrata* is able to penetrate the biological systems of the human body. Moreover, an increasing proportion of *C. glabrata* infections pose a threat to the health of patients, especially if the causes of drug resistance cannot be traced back to either genetic mutations or previous antifungal treatment. As our results indicate, echinocandin resistance was quite frequent and, in several cases, was accompanied by azole resistance. Therefore, amphotericin B and 5-flucytosine remain the only two last-resort drugs capable of fending off acute fungemia.

The selection of genes associated with antifungal drug resistance could be further expanded, and their expression at the mRNA and protein levels should be assessed for a more comprehensive evaluation of the influence of the detected mutations.

## Data Availability

Raw sequencing data was deposited in the NCBI Sequence Read Archive as bioproject: PRJNA1163087.
